# Supplementary material for: Analyses of Genetic Diversity in the Endangered “Berrenda” Spanish Cattle Breeds Using Pedigree Data
Source: Animals (Basel). 2022 Jan 20;12(3):249. doi: 10.3390/ani12030249 (PMC8833649; doi:10.3390/ani12030249)
Supplement: Supplementary file 1 [file animals-12-00249-s001.zip › Suplementary material (1).pdf]

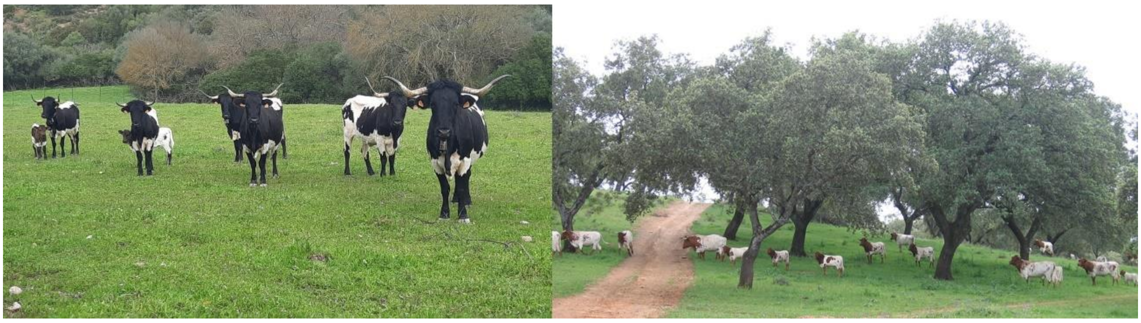

Figure S1: Herds of "Berrenda en Negro" and "Berrenda en Colorado" cattle breeds in Dehesa Ecosystem (rights owned by ANABE).

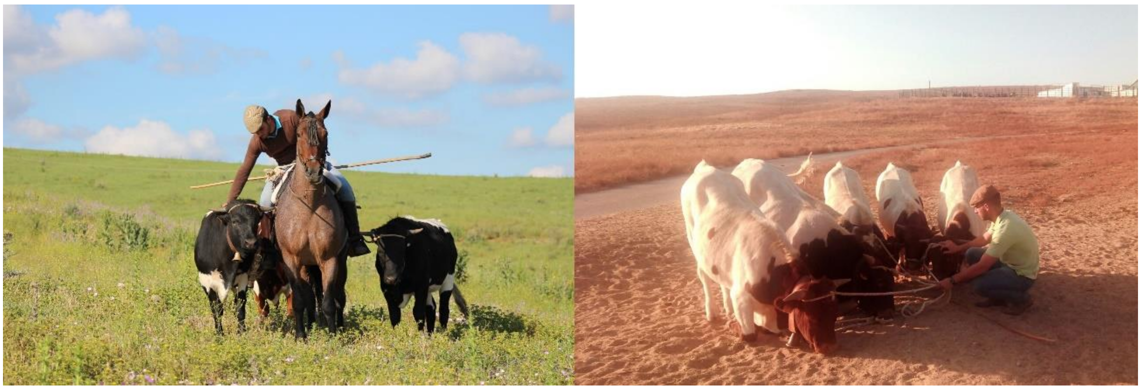

Figure S2: Training of oxen for handling of fighting Bulls (rights owned by ANABE).
